# Development of an Active Crosslinked Nanocomposite Film Based on Gelatin‐Ethyl Cellulose, Trans‐Cinnamaldehyde, and Silver Nanoparticles for the Preservation of Sliced Meat

**DOI:** 10.1111/1750-3841.70851

**Published:** 2026-01-16

**Authors:** Elham Sarmast, Shiv Shankar, Stephane Salmieri, Sahra Amel Rahmouni, Monique Lacroix

**Affiliations:** ^1^ Armand‐Frappier Health Biotechnology Research Centre, Institut National de la Recherche Scientifique (INRS), Research Laboratories in Sciences, Applied to Food (RESALA), MAPAQ Research Chair in food safety and quality Canadian Irradiation Center (CIC), International Atomic Energy Agency (IAEA) Collaborating Centre, Institute of Nutrition and Functional Foods (INAF) Laval Canada; ^2^ School of Food Science and Environmental Health College of Sciences and health Technological University Dublin Dublin Ireland

**Keywords:** bioactive films, crosslinking, gelatin, meat preservation, nanocomposite, trans‐cinnamaldehyde

## Abstract

This study aimed to develop a novel bioactive biobased packaging film combining gelatin (G) and ethyl cellulose (EC), cardanol and glycerol as plasticizers, trans‐cinnamaldehyde (TCA) as a crosslinker/bioactive compound, and silver nanoparticles (AgNPs) as bioactive agents. The degree of crosslinking, mechanical and thermal properties, water solubility (WS), and water vapor permeability (WVP) of the films were analyzed. Molecular interactions within the film matrix were determined by FTIR spectroscopy. The G‐EC film, in a 1:2 weight ratio, exhibited improved physicochemical properties compared to the pure G film, with a low WS (7.8%). The incorporation of TCA and AgNPs—to form bioactive nanocomposite films—improved the barrier and mechanical properties of the film (WS 0.33%, WVP 2.39 g·mm·m^−^^2^·day^−^^1^·kPa^−^^1^, tensile strength 9.6 MPa) with a 57% degree of crosslinking. Thermal analyses (TGA/DSC) revealed improved thermal stability of these films, with an increase in the maximum decomposition temperature (*T*_max_ up to 370°C–380°C) and the glass transition temperature (*T*_g_ 29.5°C) after incorporation of TCA and AgNPs. FTIR analysis confirmed the amide/imine covalent crosslinking reaction between TCA and G, and polymer network stabilization by hydrogen bonding between G and EC. The nanocomposite films demonstrated effective in vitro antibacterial activity against pathogenic and spoilage bacteria, with an inhibition spectrum of 27%–54%. An in situ test performed on chilled sliced meat packaged in nanocomposite films showed a significant extension of its shelf‐life, up to 9 days. These results highlight the potential of G‐EC‐TCA‐AgNPs films as a sustainable packaging solution for improved meat preservation.

**Practical Applications:**

G‐EC‐TCA‐AgNPs films developed in this study could be applied as bioactive and biobased food packaging films to extend the shelf‐life of meat products. Films are made of biopolymers and address the challenges of sustainability and circular economy. TCA and AgNPs could be used as a synergic antimicrobial combination encapsulated in films to prevent the growth of pathogenic and spoilage microorganisms in meat.

## 1 Introduction

In recent years, environmental issues related to the disposal of synthetic petrochemical polymers have been of increasing concern. In this context, packaging microplastic particles, exceeding acceptable levels, can be released into food or water and cause detrimental effects on human health, as well as the well‐being of animals and aquatic life (Horvat et al. 2022). Therefore, sustainable development policies tend to use emerging alternatives such as biobased polymers, which are mainly derived from biological or agricultural resources, waste, and food by‐products. Biodegradable polymers from different sources of protein and polysaccharides can effectively be used as food packaging (Etxabide et al. 2017).

Gelatin (G) is a protein extracted from the bones and skin of animals and is classified as a “generally recognized as safe” (GRAS) compound according to the Food and Drug Administration (FDA) regulations. This protein is used in a wide variety of applications in food, pharmaceutical, biomedical, and packaging industries (Shankar et al. 2015; Liu et al. 2018a). Edible, biodegradable, and biocompatible films made of G are interesting due to their good mechanical properties. However, water solubility (WS) and thermo‐sensitivity of G limit its application as food packaging. Blending and crosslinking of G are promising techniques to enhance the mechanical and barrier properties of the G film (Chang et al. 2012; Ahammed et al. 2021). Cellulose‐based material has gained more attention due to the vast amount of raw material resources and environmentally friendly aspects (Chen et al. 2016). Ethyl cellulose (EC) is a hydrophobic linear polysaccharide derived from cellulose containing the ethyl end groups that replaced the hydroxyl groups of cellulose (Heredia‐Guerrero et al. 2018). EC displays some properties such as non‐toxicity, thermoplasticity, flexibility, high mechanical strength, film‐forming ability, and transparency, which offers a variety of applications such as film formation, thickening agents, and microcapsules in the food and pharmaceutical industries (Horvat et al. 2022). One of the exciting characteristics of EC is its heat resistance. Moreover, composite films made of EC with other biopolymers can modify the crystalline structure of EC film and improve the mechanical, barrier, and thermal properties of the blended film (Chen et al. 2016). Biopolymers can be used to encapsulate bioactive compounds and form antimicrobial films, thus preventing direct contact of active substances with food without compromising antimicrobial efficacy (Begum et al. 2024).

Among bioactive compounds, trans‐cinnamaldehyde (TCA) is considered a GRAS substance by various authoritative organizations, including the FDA, the Flavor and Extract Manufacturers Association (FEMA), and has been approved by the Council of Europe (CoE) as a food additive (Doyle and Stephens 2019; Mahmud et al. 2024b). Considering this, TCA has been used in various commercial foods as a pleasant taste and odor enrichment. Furthermore, its antimicrobial and antioxidant activity make it a powerful agent to be successfully integrated into edible antimicrobial films, effectively inhibiting foodborne pathogens through either direct contact or the emission of vapor within sealed containers (Baskaran et al. 2010). Additionally, the aldehyde functional group within the structure of TCA has been investigated as a viable crosslinking agent for biopolymers like gliadin, chitosan, and G (Balaguer et al. 2015; Demitri et al. 2016; Liu et al. 2018a; Thongsrikhem et al. 2022).

Silver nanoparticles (AgNPs) also exhibit effective antimicrobial properties (Moosavi et al. 2025). Nanotechnology has recently emerged as one of the most innovative and promising technologies in various sectors (Mahmud et al. 2022). In this regard, AgNPs offer numerous applications in the fields of food, packaging, and medicine, owing to their distinctive characteristics, including a large specific surface area and potent antimicrobial activity coupled with high stability. In addition, AgNPs exert strong antimicrobial activity against both gram‐negative and gram‐positive bacteria. It has been reported that the practical use of AgNPs in various types of food packaging effectively inhibits microbial growth and prolongs the shelf‐life of food products (Zimoch‐Korzycka and Jarmoluk 2015; Hong et al. 2021; Sarmast et al. 2024). In accordance with European safety regulations, the migration of AgNPs into food from packaging films is limited to 0.05 mg/kg. Moreover, stabilization of nanoparticles in polymeric matrices generally exhibits minimal migration due to several factors such as food contact, polymer‐nanoparticle interactions, and environmental conditions (temperature, pH, moisture, and fats) (Syed et al. 2025).

Some studies have been carried out to evaluate the characteristics of G‐EC composite films (Liu et al. 2018c, 2018b; Wang et al. 2020; Beikzadeh et al. 2021). In this sense, a study on the combination of these 2 biopolymers, the incorporation of TCA and AgNPs as potential synergic active agents for food preservation, is relevant. Moreover, the double role of TCA as an antimicrobial agent and a crosslinker to enhance film properties is a key element for innovative research. The objective of this work was to develop a water‐resistant G‐EC nanocomposite film by the solvent casting process. The effect of adding TCA and AgNPs as bioactive agents−and TCA as crosslinker−was also evaluated on the physicochemical, mechanical, and thermal properties of the films. Finally, the antimicrobial efficacy of the bioactive films was measured in vitro as well as their ability to extend the shelf‐life of sliced meat.

## 2 Materials and Methods

G powder (Type B; bloom number: 250; average molecular weight (M_W_:100 kDa), EC (viscosity (5% in toluene/ethanol 80:20): 3–7 cP; M_W_: 20–30 kDa; ethoxyl substitution: 48.0%–49.5%), TCA (97% purity), absolute ethanol, acetic acid and trinitrobenzene sulfonic acid (TNBS) were obtained from MilliporeSigma Canada Ltd (Oakville, ON, Canada). AgNPs were provided by NanoBrand (Laval, QC, Canada). These AgNPs were stabilized in a capping agent composed of polyvinylpyrrolidone (M_W_: 40 kDa; Thermo Fisher Scientific, St‐Laurent, QC, Canada) and polyethylene glycol (M_W_: 600 Da, Thermo Fisher Scientific) at pH 6. They consisted of a mixture of 2 nanosphere fractions at 20–35 nm and 3–7 nm measured by transmission electron microscopy (TEM Hitachi H‐7100, Hitachi High‐Tech Canada Inc., Toronto, ON, Canada). Tryptic Soy Agar (TSA), Tryptic Soy Broth (TSB), Brain Heart Infusion (BHI) agar, BHI broth, Mueller Hinton broth (MHB), De Man‐Rogosa‐Sharpe (MRS) agar, cetrimide agar, and streptomycin thallous acetate actidione (STAA) agar were provided by Alpha Biosciences (Baltimore, MD, USA).

### 2.1 Microorganisms and Growth Conditions

Various bacterial strains were employed in this study. *Escherichia coli* O157:H7 ATCC 25922, *Pseudomonas aeruginosa* ATCC 27853, *Brochothrix thermosphacta* ATCC 11509, *Salmonella* Typhimurium ATCC SL1344, and *Staphylococcus aureus* ATCC 25923 were purchased from Cedarlane (Burlington, ON, Canada). Bacterial strains were stored at −80°C in a BHI medium containing 20% glycerol (v/v). Before each experiment, working cultures were prepared by subculturing 1 mL of each stock culture in 9 mL of BHI broth for *B. thermosphacta*, and TSB for the other bacteria. Cultures were incubated for 24 h at 30°C for *B. thermosphacta* and 37°C for the other species. Then the bacterial cultures were centrifuged at 2000 × *g* for 15 min at 4°C and diluted with 0.85% (w/v) saline water to obtain a working culture of approximately 10^6^ CFU/mL (established by a standard curve OD at 600 nm vs. CFU/mL).

### 2.2 Minimum Inhibitory Concentration (MIC) and Fractional Inhibitory Concentration Index (FIC) of TCA and AgNPs

The MIC assessment of the antimicrobial compounds against both spoilage and pathogenic bacteria was conducted in sterile 96‐well microplates (Sarstedt, Saint‐Leonard, QC, Canada) using the microdilution method outlined by Sarmast et al. (2023). A 100 µL aliquot of 2‐fold serial dilution (from 10 to 0.01 µL/mL) of active agent (TCA or AgNPs suspension) was deposited in a 96‐well microplate. Each well was then inoculated with 100 µL of bacterial suspension (BHI for *B. thermosphacta* and MHB for other bacteria) at a concentration of 10^6^ CFU/mL, making a final volume of 200 µL and a final bacterial concentration of 5 × 10^5^ CFU/mL in each well. Afterwards, the microplates were incubated under aerobic conditions and agitated at 80 rpm for 24 h (at 30°C for *B. thermosphacta* and 37°C for other bacteria). Then, the absorbance was measured at 595 nm in a BioTek ELx800 absorbance microplate reader (BioTek Instruments Inc., Winooski, VT, USA). The MIC was determined as the lowest concentration of the active agent, showing equal absorbance as the negative control and demonstrating the complete growth inhibition of microorganisms.

The checkerboard method, as outlined by Mahmud et al. (2023) was employed to determine the FIC index of combined TCA and AgNPs and to verify possible synergistic effects against bacteria. Each well on the microplate represented a distinct combination of the 2 antimicrobial agents. The first active agent (50 µL of TCA) was distributed in binary dilutions along the X‐axis, while the second one (50 µL of AgNPs suspension) followed binary dilutions along the Y‐axis. Therefore, in each well, the final volume comprised 100 µL, consisting of 50 µL of each antimicrobial dilution. Subsequently, 100 µL of a bacterial suspension (containing 6 log CFU/mL of the target bacteria) was added to the wells to obtain a final volume of 200 µL. The plates were then incubated as described for MIC measurements, and the absorbance was measured at 595 nm. The FIC index was computed by Equation 1 and Equation 2:

(1) $$\mathrm{FIC}\;=\;\mathrm{FIC}\left(A\right)+\mathrm{FIC}\left(B\right)$$

(2) $$\begin{array}{c} \mathrm{FIC}\;\left(A\right)=\frac{\mathrm{MIC}\left(A\right)\mathrm{combined}}{\mathrm{MIC}\left(A\right)\mathrm{alone}}\;\text{and}\;\mathrm{FIC}\;\left(B\right)=\frac{\mathrm{MIC}\left(B\right)\mathrm{combined}}{\mathrm{MIC}\left(B\right)\mathrm{alone}} \end{array}$$

Synergistic effect: FIC < 0.5; additive effect: 0.5 ≤ FIC ≤ 1; non‐interactive (indifferent) effect: 1< FIC ≤ 4; and antagonistic effect: FIC > 4.

### 2.3 Film Preparation by Solvent Casting Process

Different weight ratios of G and EC (2:1, 1:1, and 1:2 w/w for G‐EC‐1, G‐EC‐2 and G‐EC‐3 films, respectively) were dissolved in 15 mL ethanol and 35 mL acetic acid to form a total concentration of 5% (w/v). After determining the optimal weight ratio for achieving a film with minimal WS and satisfactory mechanical properties, TCA and the TCA‐AgNPs mixture were incorporated into the film‐forming solution. TCA was added at a concentration of 5% (w/w, dry weight of G) for the G‐EC‐4 film. The mixture of thTCA‐AgNPs (ratio 1.5:1 v/v, pure liquid TCA/liquid suspension of AgNPs in capping agent) was also added at a concentration of 5% (w/w, dry weight of G) for the G‐EC‐5 film. Film solutions were stirred at 45°C for 3 h in a closed system to ensure the complete dissolution of polymers. Glycerol 30% (v/w, dry weight of G) and cardanol 20% (v/w, dry weight of EC) were added as plasticizers to the polymer solution. A quantity of 25 mL of film solution was poured into Teflon plates and dried at 25°C and 50% relative humidity (RH) for 24 h. Films were peeled off and kept in a desiccator at 21°C/56% RH for at least 48 h prior to analyses. Digital images of G‐EC‐3 and G‐EC‐5 films are shown in Supplementary data, Figure 1.

### 2.4 Degree of Crosslinking by the TNBS Assay

The degree of crosslinking was determined according to the method of Sarmast et al. (2024) by comparing the amount of reacted primary amino groups in G (i.e., crosslinked film) to the total free primary amino groups (i.e., control film), expressed as a percentage. Two (2) concentrations of TCA (3 and 5% w/w) were used as crosslinkers. A piece of film (11 mg/replicate) was maintained in an alkaline solution composed of 1 mL of 0.5% (w/v) TNBS and 1 mL of 4% (w/v) of sodium bicarbonate at 40°C for 2 h, reacting into a TNBS complex of primary amines. Then the film was neutralized and hydrolyzed using 3 mL of 6 M HCl at 60°C for 90 min, producing a trinitrophenyl derivative. After extracting non‐reactive reagents, the sample was diluted, and its absorbance was measured at 346 nm using a Cary 8454 UV‐Visible spectrophotometer (Agilent Technologies Canada Inc., Saint‐Laurent, QC, Canada). The experiment was done in triplicate (n = 3). The degree of crosslinking was calculated by using Equation **3**:

(3) $$\mathrm{Degree}\;\mathrm{of}\;\mathrm{crosslinking}\;\;\left(\%\right)=\left(1-\frac{\frac{A_{S}}{W_{S}}}{\frac{A_{C}}{W_{C}}}\right)\;\times \;100$$

A_S_: absorbance of sample (crosslinked film); W_S_: weight of sample (crosslinked film); A_C_: absorbance of control; and W_C_: weight of control.

### 2.5 Water Resistance of Films

#### 2.5.1 Water Solubility (WS)

WS of films was defined as the dry mass content of films that were solubilized after 24 h of immersion in water at 25°C (cold, namely WS_C_) and 100°C (hot, namely WS_H_) according to the methods described by Sarmast et al. (2024). Pre‐cut films (2 × 2 cm) were dried completely at 110°C, weighed, and then immersed separately in 100 mL of water at 25°C ± 2°C and 100°C ± 2°C for 24 h. After that, the residual film was removed and dried at 70°C for 24 h. The final weight of the film was measured, and WS (%) was calculated with Equation 4:

(4) $$\mathrm{WS}\;\;\left(\%\right)=\frac{W_{0}-W_{1}}{W_{0}}\;\times \;100$$

*W*_0_: initial weight of the film and *W*_1_: weight of the residual, undissolved part of film.

#### 2.5.2 Water Vapor Permeability (WVP)

WVP analyses of the films were performed according to the ASTM E96/E96M – 16 method (**ASTM** 2018) with some modifications. Film specimens were pre‐conditioned at 25°C/57.6% relative humidity (RH) for 48 h, in a desiccator containing a saturated solution of sodium bromide. Film thickness was measured at 5 random positions using a standard Mitutoyo 543–792 B Digimatic Indicator (model ID‐S112EX; BDI Canada Inc., Laval, QC, Canada) with a resolution of 0.001 mm. Films were sealed onto Vapometer EZ‐cups (model 68–3000; Thwing‐Albert Instrument Company, West Berlin, NJ, USA) containing 30 g of dried calcium chloride. The assemblies were kept at 25°C and 60% RH in a Shellab 9010L humidity chamber (Sheldon Manufacturing Inc., Cornelius, OR, USA). Samples were weighed periodically by calculating the constant rate of change (linear slope) when steady state was reached. Tests were stopped before the desiccant exceeded 10% of its starting weight. WVP (g·mm·m^−^^2^·day^−^^1^·kPa^−^^1^) was determined according to Equation 5:

(5) $$\mathrm{WVP}\;=\frac{\Delta w\;\times T}{t\;\times \;A\;\times \Delta P}$$

∆*w*: weight gain (g) from the straight line; *T*: film thickness (mm); *t*: time during which ∆w occurred (day); ∆*w/t*: slope of the straight line; A: film surface area (31.67 × 10^−4^ m^2^); ∆P: differential vapor pressure (∆P  =  3.2823 kPa at 25°C/60% RH).

### 2.6 Mechanical Properties of Films

Mechanical properties of the films were measured by a Universal Testing Machine (UTM) H5KT (Tinius Olsen, Horsham, PA, USA), equipped with a 100 N‐load cell and 1.5 kN‐specimen grips, based on the ASTM D638‐99 method (2015). Film specimens were pre‐conditioned at 25°C/57.6% relative humidity (RH) for 48 h in a desiccator containing a saturated solution of sodium bromide. The thickness of the film was measured by a 543–792 B Digimatic Indicator at 5 random positions. Film width was measured using a Traceable caliper (resolution: 0.1 mm; accuracy: ± 0.2 mm; Thermo Fisher Scientific). Film specimens were conformed to a length of 60 mm and a width of 12 mm or to Type IV dimensions with a die cutter before testing. Tests were performed at 23°C ± 2°C and 50% ± 5% RH with at least 5 specimens for each sample (n = 5). Test parameters were set up for “Tensile from position” test type with the following selections: 25 mm effective gauge length, flat specimen shape, 1 number of entries, and minimum type. The position rate of machine control was fixed to 50 mm/min. The Y‐ and X‐axes were assigned to load (100 N‐range) and position (500 mm‐range) coordinates, respectively. Tensile strength (TS, MPa), tensile modulus (TM, MPa), and elongation at break (Eb, %) were determined after rupture of the film under the effect of elongation, using Horizon software (ver. 10.3.1.10).

### 2.7 Thermal Properties of Films

#### 2.7.1 Thermogravimetric Analysis (TGA)

TGA analysis was carried out by using a TGA 4000 thermogravimetric analyzer (PerkinElmer, Woodbridge, ON, Canada). Samples (10–20 mg) were heated from 30°C to 600°C at a heating rate of 10°C/min under a dynamic nitrogen atmosphere (20 mL/min). The 1st derivatives of TGA curves (DTG curves) were plotted to determine differential TGA values. The maximum decomposition temperature (*T*_max_) of films was determined from the DTG curves, while the char yield, the residual mass at 600°C, and the weight loss (%) were measured from the TGA curves (Shankar and Rhim 2018).

#### 2.7.2 Differential Scanning Calorimetry (DSC)

DSC analysis was performed using a DSC 4000 calorimeter (PerkinElmer) to investigate the glass transition of films. Pre‐conditioned film samples (5–10 mg) were sealed hermetically in a 40‐µL standard aluminum pan (PerkinElmer) and subjected to a heat‐cool‐heat cycle process under a nitrogen flow rate of 20 mL/min. Firstly, samples were heated from –70°C to 200°C at a rate of 10°C/min. Then samples were cooled from 200 to –70°C at 5°C/min. In the end, the 2nd heating cycle was performed up to 200°C at a rate of 10°C/min. The crystalline peak temperature was determined from the cooling cycle, and the glass transition temperature (*T*_g_) was determined from the midpoint of the heat flow change in the 2nd heating cycle (Benbettaïeb et al. 2016).

### 2.8 Structure Analysis of Films by FTIR Spectroscopy

The crosslinking reaction between G‐EC and TCA was analyzed with a Spectrum One spectrophotometer (PerkinElmer) equipped with an attenuated total reflectance (ATR) device and a lithium tantalate detector. Film specimens were pre‐conditioned at 25°C/57.6% RH for 48 h in a desiccator containing a saturated solution of sodium bromide. Spectra were analyzed by using Spectrum 10.7.2.1630 software. Films were placed onto a zinc selenide crystal, and the analysis was performed after background within the spectral region of 4000–650 cm^−1^, with 64 scans recorded at a 4 cm^−1^ resolution. After attenuation of total reflectance and baseline correction, spectra were normalized within the whole infrared region with the minimum transmission peak set at 75% (Liu et al. 2018b). Tests were performed at 23°C ± 2°C and 50% ± 5% RH in triplicate (n = 3).

### 2.9 Antibacterial Activity Assessment by Disk Diffusion Test

The antimicrobial activity of G‐EC‐4 and G‐EC‐5 films was evaluated in vitro by agar diffusion assay, as described by Thongsrikhem et al. (2022). The antibacterial properties against *S*. Typhimurium, *P. aeruginosa*, *B. thermosphacta*, *S. aureus*, and *E. coli* were determined. The films were cut into 1 cm diameter disc‐shaped specimens and were sterilized by UV‐C irradiation on both sides for 15 min in a biosafety cabinet. These films were then placed on agar plates previously streaked with a culture of the test bacteria. After 24 h of incubation at 37°C, the presence of a clear area around the film, indicating no microbial growth, was measured. The percentage of inhibition capacity (IC, %) was calculated according to Equation 6 (Mahmud et al. 2024a):

(6) $$\mathrm{IC}\;\left(\%\right)=\frac{\mathrm{Diameter}\;\mathrm{of}\;\mathrm{inhibition}\;\mathrm{zone}\;\left(\mathrm{mm}\right)}{\mathrm{Diameter}\;\mathrm{of}\;\mathrm{Petri}\;\mathrm{dish}\;\left(\mathrm{mm}\right)}\;\times \;100$$

### 2.10 in situ Test—Microbial Analysis of Sliced Meat

The antimicrobial activity of G‐EC‐4 and G‐EC‐5 films was evaluated in situ on sliced meat, based on a method described by Sarmast et al. (2024). Fresh sliced meat was provided by Montpak International (Laval, QC, Canada). Meat samples were divided into 3 groups for 3 types of films: G‐EC‐4, G‐EC‐5, and control (G‐EC film without antimicrobial formulation). Meat samples (10 g) were cut into rectangular shapes, and films (5 × 4 cm) were applied on each side of the meat, and then inserted in Whirl‐Pak sterile bags (supplied by Thermo Fisher Scientific). Bags were stored at 4°C, and microbiological analyses were performed on days 0, 3, 6, 9, 12, 15, 18, 21, 24, 27, and 30, as follows: meat samples (10 g) were combined with 90 mL of 0.1% peptone water and blended for 1 min at 260 rpm using a Seward 400 Circulator Stomacher (Thermo Fisher Scientific). TSA was used for the enumeration of total mesophilic flora (TMF) at 37°C for 24 h, MRS agar for lactic acid bacteria (LAB) at 30°C for 72 h, cetrimide agar for *P. aeruginosa* at 37°C for 48 h, and STAA agar for *B. thermosphacta* at 25°C for 48 h. The limit of detection for microbial enumeration was set at 10 CFU/g. Microbiological analyses ended when TMF counts reached 7 log CFU/g (meat shelf‐life limit).

### 2.11 Statistical Analysis

All data were analyzed by one‐way analysis of variance (ANOVA) using IBM SPSS Statistics software (ver. 26.0; IBM Canada Ltd, Markham, ON, Canada). The variable frequencies were checked to follow a normal distribution. Analysis of variance (ANOVA) was used to assess the differences between the means. Duncan's test was used for multiple comparisons based on variance homogeneity, and significant differences were indicated by *p* ≤ 0.05. All experiments were performed in triplicate (n = 3).

## 3 Results and Discussion

### 3.1 MIC and FIC Values of TCA and AgNPs

The MIC and FIC values for TCA, AgNPs, and their combination against a range of bacterial strains are summarized in Table 1. The MIC values of TCA ranged from 1250 to 500 ppm against the tested strains. Indeed, a high antimicrobial activity was observed against *S*. Typhimurium, *B. thermosphacta*, and *E. coli* with a concentration of 1250 ppm, while 500 ppm was required to inhibit the growth of *S. aureus* and *P. aeruginosa*. Besides, the MIC of AgNPs exhibited lower MIC values of 7.8 ppm for *S*. Typhimurium, *B. thermosphacta*, *E. coli*, *S. aureus*, and 27 ppm for *P. aeruginosa*. The FIC values of the antimicrobial formulation of TCA and AgNPs were between 0.37 and 0.62 for all the tested bacteria. The combination of TCA + AgNPs had the highest synergistic activity against *S. aureus*, having an FIC index of 0.37, whereas the rest of the tested bacteria showed an additive effect.

**TABLE 1:** MIC and FIC of antimicrobial compounds against target bacteria.

Antimicrobial		Bacteria				
		*S*. Typhimurium	*B. thermosphacta*	*E. coli*	*S. aureus*	*P. aeruginosa*
**TCA**	MIC (ppm)	1250	1250	1250	500	500
**AgNPs**	MIC (ppm)	7.8	7.8	7.8	7.8	27.0
**TCA‐AgNPs**	FIC	0.62AD	0.62AD	0.78	0.37S	0.56AD

*Note*: Activity: FIC ≤ 0.5: synergistic effect (S); 0.5 < FIC ≤ 1: partial synergism/additive (AD); 1 < FIC ≤ 4: no interaction (NI); FIC > 4: antagonistic effect (AG).

There are several mechanisms for the antimicrobial activity of TCA (Doyle and Stephens 2019). TCA has been identified as an inhibitor of the cell division process by binding to filamentous temperature‐sensitive protein Z (FtsZ). FtsZ, a crucial cytoplasmic protein involved in cell division, is found in the cell walls of various bacteria, including *E. coli*. Research has revealed that FtsZ forms a ring‐shaped structure known as a Z‐ring at the cell's center. This dynamic Z‐ring plays a vital role in facilitating cytokinesis and sequentially recruiting other proteins related to cell division. As a result, the Z‐ring contracts, leading to the formation of a septum and ultimately resulting in the creation of two daughter cells. Inhibition of FtsZ can prohibit bacterial division and proliferation. The inhibitory effects of TCA on biofilm formation by various bacteria such as *E. coli*, *Pseudomonas* spp., and *S*. Typhimurium have been studied (Kavanaugh and Ribbeck 2012; Silva et al. 2018). The antibiofilm activity of TCA can be attributed to the presence of the aldehyde functional group in its chemical structure. Rao et al. (2019) highlighted how the disruption of cell membrane permeability leads to bacterial inactivation by allowing the infiltration of ions and organic molecules from the external environment. This disruption ultimately results in the loss of membrane integrity, which is vital for the survival of microbial organisms.

Moreover, when the cell membrane is disrupted, certain intracellular components are released. The bacterial plasma membrane facilitates the passage of small ions like K^+^ and Na^+^, which are essential electrolytes required for various cell membrane functions and normal enzymatic activities (Diao et al. 2014). Maintaining ion balance is crucial for preserving the cell's energy status and is essential for membrane‐coupled, energy‐dependent processes, including metabolic regulation, solute transport, turgor pressure control, and mobility. Consequently, even minor alterations in membrane structure and integrity can have detrimental effects on cell metabolism, ultimately leading to cell death. TCA can cause a substantial reduction in intracellular ATP levels. Gill and Holley (2006) reported that the introduction of 10 mM TCA to *E. coli* and 40 mM TCA to *L. monocytogenes* resulted in notable reductions in intracellular ATP levels.

Lipophilic compounds, such as TCA, can have indirect effects on the levels of adenine nucleotides in cells by influencing processes involving proton motive force and phosphate potential, often mediated by F0F1 ATPases. One prominent adenine nucleotide affected by these mechanisms is ATP. The observed stimulation of proton and/or ion‐translocating ATPases by lipophilic compounds might stem from their ability to disrupt the proton motive force, which subsequently limits ATP hydrolysis. Consequently, when the normal function of ATPases is interrupted, it can perturb the cell's electrolyte balance, directly impacting cellular homeostasis. This disruption can lead to a significant decrease in ATP levels, and in certain cases, it can even trigger cell death if it severely affects cellular respiration and overall metabolic processes. This highlights the complex interplay between lipophilic compounds, cellular energetics, and the maintenance of cellular functions (Rao et al. 2019).

The positive charge exhibited by AgNPs and the negative charge of the bacterial cell membrane create an electrostatic attraction, thus promoting the attachment of NPs onto the membrane (Abbaszadegan et al. 2015). The antimicrobial mechanism of AgNPs is notably linked to their accumulation on the bacterial cell wall, damaging the membrane by various irregularly shaped protrusions. This process thus creates breaches that affect the integrity of the outer membrane and cause the release of cellular constituents. Furthermore, AgNPs can release Ag+ ions, contributing to cellular degradation by their oxidative dissolution in the presence of oxygen. Finally, the interaction of Ag+ ions with the thiol groups of enzymes and proteins of the cell wall leads to instability of these bonds. This interaction has multiple consequences: altering the tridimensional structure of proteins, blocking active binding sites, altering the transport and release of potassium ions, hindering the synthesis of adenosine triphosphate (ATP) (Tang and Zheng 2018), and ultimately leading to the degradation of DNA (Mahmud et al. 2022).

The synergistic combination of essential oils (EOs) with NPs provides a strategy for effectively controlling microbial growth. This combined approach targets various cellular levels, initiating the changes in the outer membrane in Gram‐negative bacteria and the cell wall in Gram‐positive bacteria. This interference disrupts the normal intra‐ and extracellular transportation and leakage of bacterial electrolytes, nutrients, and enzymes. Upon penetrating the bacterial cell, the active compounds derived from EOs and NPs interact with proteins and enzymes, leading to the complete inactivation of the bacteria (Rai et al. 2017). This synergetic and additive activity of antimicrobial agents presents a promising application for microbial control.

### 3.2 Degree of Crosslinking

The evaluation of free amino groups that remained unlinked in the composite film was compared to those in non‐crosslinked G and expressed as the degree of crosslinking. As a result, the degree of crosslinking for G‐EC‐TCA 3% and G‐EC‐TCA 5% was approximately 33% ± 2.4% and 57% ± 1.8%, respectively. Therefore, G‐EC‐TCA 5% was selected for crosslinking further composite films. Chemical reactions between G and TCA are based on the Schiff base reaction, involving the bonding of the amino group of G with the aldehyde group of TCA and resulting in imine group formation (C = N), as well as the Michael reaction, which forms bonds between the amino groups of G and the α,β‐unsaturated group present in TCA. Thongsrikhem et al. (2022) obtained a 44% degree of crosslinking for the G with bacterial cellulose film crosslinked with TCA.

### 3.3 Water Resistance of Films

The WS and WVP of films are presented in Table 2.

**TABLE 2:** Water resistance and mechanical properties of the films.

Film	Thickness (mm)	WS_C_ (%)	WS_H_ (%)	WVP (g·mm·m^−^^2^·day^−^^1^·kPa^−^^1^)	TS (MPa)	TM (MPa)	Eb (%)
G‐EC‐1	0.249 ± 0.069^d^	40.74 ± 0.80^e^	51.06 ± 0.73^e^	5.50 ± 0.48^d^	5.42 ± 0.26^a^	32.51 ± 1.72^a^	119.42 ± 2.72^c^
G‐EC‐2	0.220 ± 0.02^d^	17.60 ± 1.42^d^	25.49 ± 1.73^d^	5.25 ± 0.22^d^	5.23 ± 0.65^a^	123.0 ± 10.84^b^	70.51 ± 6.98^b^
G‐EC‐3	0.169 ± 0.013^b^	7.84 ± 0.62^c^	11.39 ± 1.01^c^	4.27 ± 0.13^c^	8.08 ± 0.96^b^	237.67 ± 23.91^c^	8.17 ± 1.47^a^
G‐EC‐4	0.197 ± 0.063^c^	1.32 ± 0.057^b^	5.44 ± 0.02^b^	3.26 ± 0.21^b^	8.76 ± 0.46^b^	261.97 ± 71.45^d^	121.92 ± 12.39^c^
G‐EC‐5	0.135 ± 0.018^a^	0.33 ± 0.17^a^	1.53 ± 0.26^a^	2.39 ± 0.11^a^	9.59 ± 0.44^c^	256.6 ± 76.31^d^	110.53 ± 8.75^c^

*Note*: Values means ± standard error. Within each column, means with different lowercase letter are significantly different (*p* ≤ 0.05).

#### 3.3.1 WS of Films

A significant advantage of G is its high WS due to the hydrophilic characteristics of polar peptides of G, which facilitates easy handling during the preparation of gels and films. However, for food packaging applications, the packaging structure must remain stable under humid conditions for a certain duration. Therefore, the WS of G becomes a critical factor in its suitability for food packaging. In this study, G‐EC films with different weight ratios were prepared. In our previous study, we found that the WS_C_ and WS_H_ of pure G film were 57.93% and 100%, respectively (Sarmast et al. 2023). The WS was significantly decreased from G‐EC‐1 and G‐EC‐3, with an important variation from 40.74% to 7.84% in WS_c_ and from 51.06% to 11.39% in WS_H_, respectively. This also shows that the combination of G and EC significantly decreased the WS of the pure G film (*p* ≤ 0.05). Besides, these results demonstrated that with increasing the weight ratio of EC to G (from G‐EC‐1 to G‐EC‐3), the WS_C_ and WS_H_ decreased significantly (*p* ≤ 0.05), which can be attributed to the tightly packed structure of EC. For this reason, G‐EC‐3 was used as a film matrix to incorporate TCA and AgNPs. Liu et al. (2018c) reported that, as the EC content increased in G‐EC nanofiber, the fibers maintained their fibrous morphology, suggesting superior water stability attributed to EC. The addition of TCA as a crosslinker and AgNPs into the film of G‐EC‐3 (weight ratio 1:2) led to a significant decrease in WS_C_ and WS_H_ in the resulting G‐EC‐5 film, with values of 0.33% and 1.53%, respectively (*p* ≤ 0.05). Therefore, G‐EC‐5 film exhibited the highest stability in cold and hot water due to its higher degree of crosslinking (*p* ≤ 0.05). The crosslinking step led to strong chemical interactions between G and TCA through Schiff base and Michael addition reactions and confirmed by FTIR analysis. The presence of these intramolecular bonds within the films restricts the accessibility of G's amino and hydroxyl groups for interaction with water (Sarmast et al. 2023). Thongsrikhem et al. (2022) reported the interaction of bacterial cellulose and G helped the WS of the composite film for up to 7 days; in addition, G film crosslinked by 3% (w/w) TCA had 3.9% WS. Shahbazi and Shavisi (2018) have developed a nanocomposite bioactive film based on chitosan, cellulose nanoparticles (CNPs), and ethanolic propolis extract (EPE) for a packaging application on minced beef meat. Although they prepared the films according to the same process used in our study (solvent casting), they did not evaluate their solubility (only their swelling index) which is a critical factor to prevent high water content in contact with meat. However, they showed that the incorporation of CNPs and EPE had a synergistic effect on the reduction of swelling index, suggesting an increase of film hydrophobicity but also intermolecular interactions between the film matrix and phenolic compounds of EPE and CNP intercalation in the film network.

#### 3.3.2 WVP of Films

WVP measurement was conducted to assess the ability of the films to act as a barrier against moisture. In food packaging, it is desirable to have a low WVP value to minimize or reduce the transfer of moisture between the packaged food and the surrounding environment, thereby preserving the freshness of the food. The WVP of the composite films of G‐EC and the incorporation of TCA and AgNPs are shown in Table 2. The G film exhibited a considerably high WVP value of 53.20 g·mm·m^−^^2^·day^−^^1^·kPa^−^^1^ due to its hydrophilic nature (Sarmast et al. 2024). The high WVP value of G film indicates a greater susceptibility to moisture absorption, which in turn impacts the mechanical characteristics of the film by inducing swelling, softening, and a decrease in mechanical strength. Consequently, the moisture levels present in the food product packed with G film can significantly affect the microbial and organoleptic qualities of food. Among the films without TCA, the WVP value of the G‐EC‐3 film was 4.27 g·mm·m^−^^2^·day^−^^1^·kPa^−^^1^, which is significantly lower than the films with an equal or lesser amount of G (G‐EC‐1 and G‐EC‐2 films) with corresponding values of 5.50 and 5.25 g·mm·m^−^^2^·day^−^^1^·kPa^−^^1^, respectively (*p* ≤ 0.05). Similarly, the WVP value for the G‐EC‐5 film containing TCA‐AgNPs was 2.39 g·mm·m^−^^2^·day^−^^1^·kPa^−^^1^, which is significantly lower than all the films (*p* ≤ 0.05). It could be assumed that the presence of a high amount of EC in the composite film changed the water vapor barrier properties and caused a decrease in WVP (Wang et al. 2020). The tight packing and the presence of inter‐ and intramolecular hydrogen bonding between the hydroxyl groups of EC and the amino groups of G led to the formation of a dense structure with low porosity in film. This dense structure hindered the permeation of water vapor through the film, resulting in reduced WS and WVP values. These findings align with a previous study where the addition of a high amount of carboxymethyl cellulose led to a decrease in the WVP of G film (Mu et al. 2012). Additionally, the dense structure of G‐EC‐4 and G‐EC‐5 film due to crosslinking of G and TCA and the presence of AgNPs resulted in the decreased migration of water through the films. NPs such as AgNPs can improve the moisture barrier properties of biopolymer films by creating a complex path that slows moisture permeation. This “tortuous path” effect extends the diffusion route, reducing the WVP and enhancing the overall barrier properties of the biopolymer (Mahmud et al. 2022).

Similarly, in carboxymethyl cellulose‐G film, an increase in EO content (2% *Mentha longifolia* L.) reduced the WVP values from 2.6 to 2.0 × 10^−10^ g·m·m^−2^·s^−1^·Pa^−1^ (Shahbazi et al. 2021). Previous studies have reported that blending hydrophobic polymers with hydrophilic polymers led to a decrease in the WVP of film. Fabra et al. (2015) reported that the hydrophobic nature of polyhydroxybutyrate (PHB) in a film of wheat gluten‐PHB decreased the WVP value. Ji et al. (2018) reported that blending 2% (w/w) PLA with G resulted in 24.2% lower WVP compared to pure G film.

Besides, the WVP decreased when hydrophobic compounds and plasticizers were used in the film, which is due to the hydrophobic nature of cardanol and TCA. The hydrophilic cardanol molecule can facilitate the uniform dispersion of EC molecule blocks within the G matrix, thereby impeding WVP. However, this decrease in water affinity can be beneficial to preserving the moisture content of fresh meat when the film is employed in food packaging (Ji et al. 2018). The water absorption of G films is mainly due to the formation of hydrogen bonds between water molecules and polar groups of G, such as OH and NH (Tyuftin et al. 2022). Therefore, crosslinking of G film limited hydrogen bonding and water absorption as well as water vapor permeation, which results in lower WVP (Zhang et al. 2019). Consequently, the hydrophilic properties of the films were mitigated, making them more effective for food packaging applications where moisture control is essential.

### 3.4 Mechanical Properties of Films

The mechanical properties (TS, TM, and Eb) of films are presented in Table 2. The effect of blending the composition of G, EC, and TCA as a crosslinker and AgNPs as antimicrobial nanofillers on the mechanical and barrier properties of the G‐EC films was evaluated. Thickness, TS, TM, and Eb values of composite films are presented in Table 2. Mechanical properties of films are important characteristics since they can ensure the film's integrity and protect it from defects (Sun et al. 2014). These properties indicate the applicability and performance of film potentially suitable for meat preservation. The mechanical properties of G film can vary depending on several factors, including the G source, extraction method, processing conditions, the addition of other polymers, and the presence of any crosslinking agents (Fernandes et al. 2022).

Regarding only G‐EC films without antimicrobial and crosslinker (G‐EC‐1, G‐EC‐2, G‐EC‐3), the thickness of the G‐EC‐3 films (higher content of EC) was lower compared to the other ones. This phenomenon might be due to their effective integration of EC with G. The TS of G film is relatively low compared to other synthetic polymers (Fernandes et al. 2022). However, the strength can be improved by crosslinking G molecules. Incorporating crosslinkers or polymers can enhance the mechanical properties of G composite, increasing its tensile strength and resistance to deformation. The TS of G‐EC‐1 to G‐EC‐3 films was between 5.23 and 8.08 MPa, and among these values, the highest TS was obtained for the G‐EC‐3 film (8.08 MPa) with higher content of EC, while the G‐EC film containing TCA and TCA‐AgNPs had TS values of 8.76 and 9.59 MPa, respectively (*p* ≤ 0.05). A similar trend was recorded for the TM value of the films. The highest value was 261.90 and 256.6 MPa for G‐EC‐4 and G‐EC‐5, respectively (*p* ≤ 0.05). This behavior may be due to the interactions between the amino groups of the G backbone and the hydroxyl groups of TCA. This increases the structural cohesion of the film and helps the film to be more resistant to physical pressures. Besides, adding AgNPs to biopolymer film strengthens its structure by filling spaces between polymer chains. This densifies the matrix, enhancing mechanical strength and reducing the elasticity of the film (Mahmud et al. 2022). Sun et al. (2014) reported that 0.5%–1% (w/v) gallic acid in chitosan films significantly increased the TS value of the film. In another study, Thongsrikhem et al. (2022) reported that the addition of bacterial cellulose and TCA to G film enhanced the TS of the film compared to G film. The presence of hydroxyl groups in EC may form weaker interactions with the Amide I of G, as indicated by the shift to a lower wavenumber in the FTIR analysis (Figure 3). It has been explained that the incorporation of NPs led to enhanced mechanical properties due to improved crystallization and the establishment of interactions between polymers and NPs (Amjadi et al. 2020). The TS of EC films has been reported to be 97 MPa. In a similar context of study, Goudarzi et al. (2023) developed k‐carrageenan‐poly(vinyl alcohol) (KC‐PVA) electrospun fiber mats used as a packaging material. The mats contained plum extract (3%) and epigallocatechin gallate (EGCG; 5–10 µg/mL) to enhance the shelf‐life quality of raw meat. Such a bioactive fiber network was characterized by high intermolecular hydrogen bonds from FTIR analysis. They observed that the incorporation of bioactive extracts into fibers enhanced their ductility but decreased the tensile resistance (due to promoted movement of molecules in the polymer network and in the absence of a crosslinker). They also noted a higher water resistance of fibers (WVP, WS, and moisture content) due to a denser and more hydrophobic polymer matrix. As previously mentioned, the presence of hydroxyl groups on the EC polymer chain is believed to contribute to the formation of strong inter‐ and intramolecular hydrogen bonds, resulting in exceptionally high TS.

The results indicated that there was a significant difference in Eb among all the films. The highest and lowest values of Eb belong to the G‐EC containing TCA (121.92%) and G‐EC‐3 (8.17%), respectively. This suggests that the presence of TCA could enhance the Eb of the film. Most results reported by other researchers indicate that crosslinking leads to less elastic film and decreases mobility (Gioffrè et al. 2012; Grover et al. 2012), due to inter‐molecular hydrogen bonding between protein and crosslinker. In our case, the addition of TCA significantly increased the Eb of G‐EC‐4 and G‐EC‐5 films, which might be because of the plasticizing ability of TCA (Sun et al. 2014). Previous studies have reported that TCA acts as a plasticizer in materials such as G‐bacterial cellulose (Thongsrikhem et al. 2022), polylactide (Ahmed et al. 2016), and chitosan (López‐Mata et al. 2018), resulting in improved Eb of the films.

### 3.5 Thermal Properties of Films

#### 3.5.1 TGA Analysis

**FIGURE 1:**
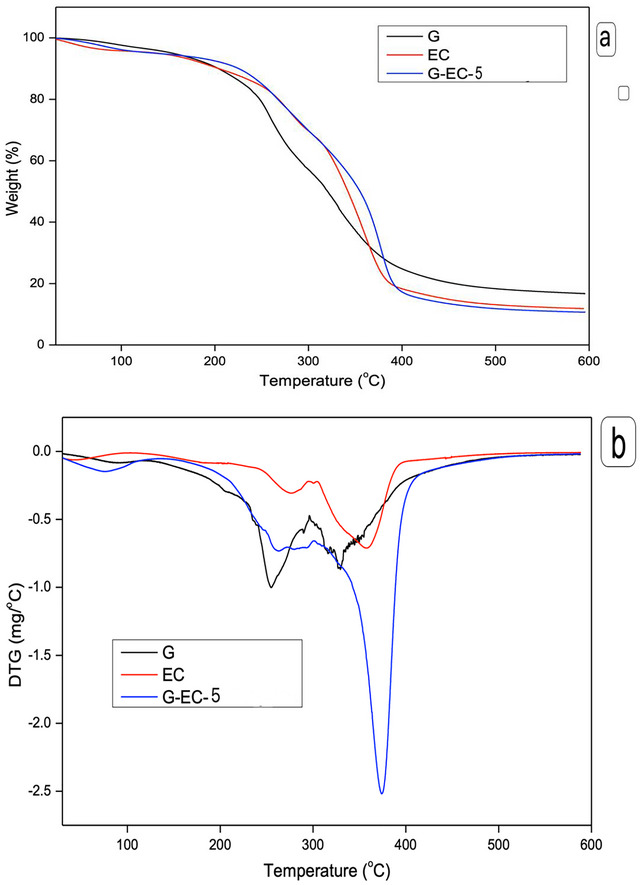
(a) TGA and (b) DTG curves of G, EC, and G‐EC‐5 composite films.

The thermal stability of the composite films was assessed through TGA analysis, and the resulting TGA and DTG curves are depicted in Figure 1. All the films exhibited multiple stages of thermal decomposition in their TGA curves (Figure 1a). Initially, there was a weight loss observed around 90°C, attributed to the removal of moisture related to the hydrophilic groups of the films, which accounted for approximately 10%–12 % of the initial weight (Shankar et al. 2015). The degradation temperature varied depending on the film type. The 2nd degradation temperature, between 220°C and 280°C and observable on all films, was mainly due to the degradation of glycerol, as observed by Shankar et al. (2016). The 3rd thermal degradation, due to the decomposition of polymers (El‐Hefian et al. 2012), showed a *T*_max_ of ∼ 335°C for G film and 370°C–380°C for EC and G‐EC‐5 films (Figure 1b). The addition of TCA and AgNPs in composite films contributed to the thermal stability of the composite films. It has been reported that the addition of NPs to the G matrix resulted in acting as a thermal barrier that restricted the release of volatile products generated during thermal decomposition (Arfat et al. 2017). After the final thermal decomposition step, the char percentages at about 600°C of G, EC, and G‐EC‐5 films were 16.7%, 11.8%, and 10.7%, respectively. Thus, it was noted that the residual percentage of EC was lower than that of G, which was further decreased by the addition of TCA and AgNPs.

#### 3.5.2 DSC Analysis

**FIGURE 2:**
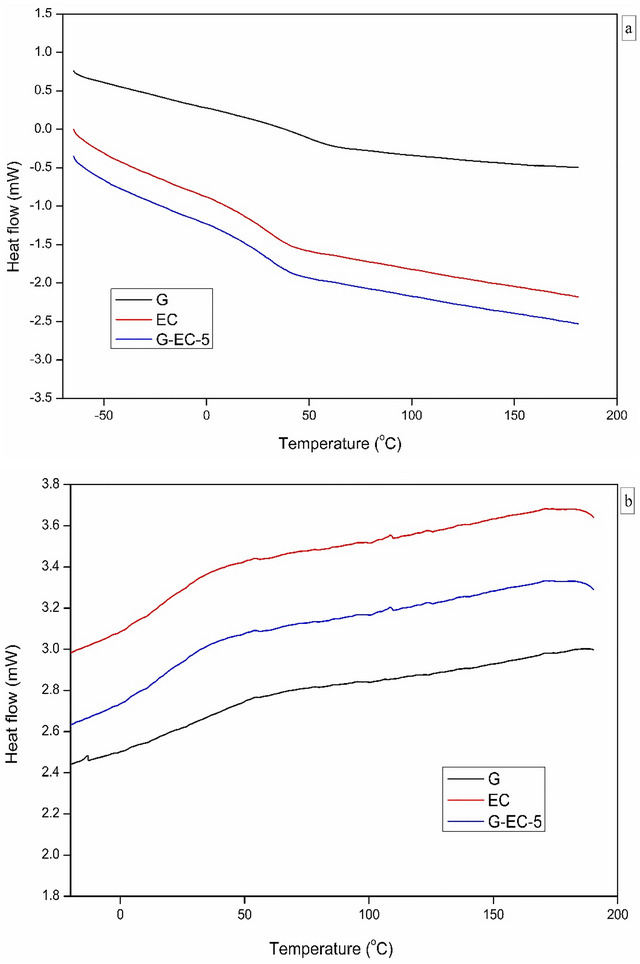
DSC curves of G, EC and G‐EC‐5 composite films, showing their *T*g at 2nd (a) heating cycle and (b) cooling cycle.

The glass transition temperature (*T*g) is a vital thermal property of polymers. *T*g is a parameter where the molecular motions of polymers start (i.e., it represents the temperature range where a polymer transitions from a glassy state, characterized by rigidity, to a rubbery state, characterized by high flexibility). The specific *T*_g_ value depends on the molecular arrangement, degree of crosslinking, and crystallinity level. *T*_g_ serves as an essential parameter for characterizing polymers, as it directly relates to their mechanical properties (Pino‐Ramos et al. 2021). The *T*_g_ values of G, EC, and G‐EC‐5 composite films were around 43.5°C, 23.0°C, and 29.5°C, respectively (Figure 2a). The *T*_g_ values of the control films (G, EC) were lower compared to those reported in previous studies (Baghi et al. 2024; Mahnaj et al. 2011; Qiao et al. 2017), possibly due to the well‐known plasticizing effect of plasticizers and moisture, which leads to a decrease of *T*_g_ (Liu et al. 2024; Qiao et al. 2017).

Moreover, the *T*_g_ value increased after the addition of TCA‐AgNPs compared to the EC film, which might be due to the crosslinking of polymer with TCA. During crosslinking, film flexibility and void volume decreased, thus increasing the *T*_g_. The reduction of endothermic peaks during the 2nd heating cycle, compared to the 1st heating cycle (not shown), indicates the destruction of the crystalline area. During the cooling step, biopolymers generally become amorphous, thus causing a loss of the helix‐coil transition, as observed by Jaiswal et al. (2024). The *T*_g_‐related DSC peaks observed after the 1st heating cycle are related to a partially crystalline G structure (Figure 2a). The weak crystalline peaks around 40°C were observed for all composite films and were mostly due to the partially crystalline polymers (Figure 2b).

### 3.6 FTIR Analysis

**FIGURE 3:**
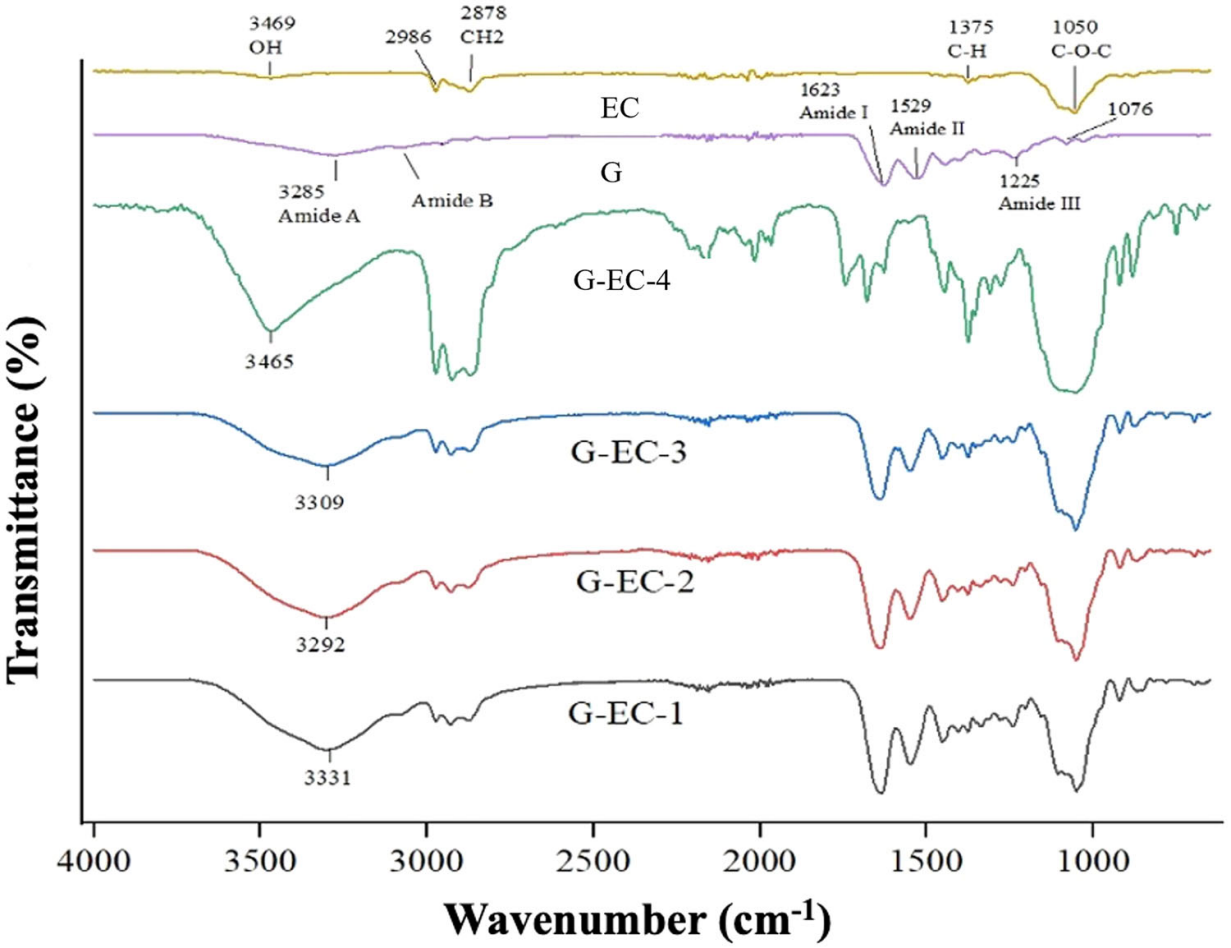
FTIR spectra of G‐EC films incorporated by TCA, and their relative G and EC controls.

Figure 3 illustrates the FTIR spectra of pure EC, pure G, and their composite films G‐EC‐1, G‐EC‐2, G‐EC‐3, and G‐EC‐4. The spectrum of pure EC shows typical polysaccharide vibration bands. Specifically, some major peaks were observed at 3469 cm^−1^ (OH stretching, hydrogen‐bonded), 3000–2850 cm^−1^ (C─H antisym and sym stretching, aliphatic moieties and cyclic hydrocarbon structure), 1380–1370 cm^−1^ (CH_3_ sym bending, aliphatic group of EC), and 1065–1015 cm^−1^ (C─O stretching, C─H─OH cyclic structure of EC). These bands reflect the characteristic molecular vibrations of EC (Shandilya et al. 2020). The spectrum of pure G exhibits some typical protein absorption bands: 3600–3100 cm^−1^ (Amide A related to ─OH stretching and Amide B to NH/NH_3_+ stretching), 2980–2850 cm^−1^ (C─H antisym and sym stretching vibrations), 1623 cm^−1^ (Amide I, C═O stretch), 1529 cm^−1^ (Amide II, N─H bending), and a weak band at 1225 cm^−1^ (Amide III, C–N stretch). This spectrum aligns well with previously published FTIR band assignments for G in water (Valcarcel et al. 2021) but also with G‐based films (Thongsrikhem et al. 2022; Sarmast et al. 2024).

Regarding the spectra of G‐EC films, overall, they exhibit bands combining those of pure G and pure EC, with some correlated variations depending on the G:EC ratio. The spectra of G‐EC‐1, G‐EC‐2, and G‐EC‐3 display a broad band at 3600–3200 cm^−1^, which was associated with the overlapped Amide A/Amide B bands of G and OH stretching of EC, glycerol, and cardanol. All of them imply ─OH hydrogen bonding and ─NH hydrogen bonding from proteins in the polymer network. This high frequency region is very sensitive to the relative concentration of blend components. As a result, this band is broader and higher in G‐EC‐1 film since it is composed of a major part of G proteins (ratio G:EC 2:1). A similar behavior was observed in the Amide I, Amide II, and, to a lesser extent, Amide III bands, as these bands are also involved in hydrogen bonding of biopolymer networks. Furthermore, this decrease in intensity of the amide bands is recognized as an indicator of a diminished helical conformation in the G chain structure, as a helix‐to‐coil transition (Liu et al. 2018b). Moreover, a shift towards lower frequencies was observed when increasing the EC concentration (from G‐EC‐1 at 3331 cm^−1^ to G‐EC‐3 at 3309 cm^−1^), suggesting a higher stability of the polymer network via hydrogen bonding. The same findings were also reported by Liu et al. (2018c), who found that the OH stretching was shifted to lower wavenumbers by increasing the weight ratio of EC. Overall, the FTIR results provided strong evidence for the presence and successful integration of both EC and G in the formulated films, resulting in a stabilized semi‐interpenetrating polymer network.

Considering the effect of TCA as a crosslinker, the spectrum of G‐EC‐4 film was very different. A broader and more intense band was observed at 3700–3200 cm^−1^ with a peak at 3465 cm^−1^, relative to the presence of TCA. This peak could be due to the presence of impurities (3% present in the specification sheet of the manufacturer), including various alcohols and low content of proteins, as reported by Lixourgioti et al. (2022). The broad band at 3010–2850 cm^−1^ is also more intense compared to the spectra of G‐EC films. This band combines C‐H stretching vibrations with the following peaks: 3008 cm^−1^ ascribed to ═C─H stretch of aromatic hydrocarbons, 2925 cm^−1^ ascribed to C─H stretch of trisubstituted alkenes (>C ═ C─H in the cyclic‐linear junction of the TCA structure), and 2854 cm^−1^ ascribed to >C─H stretch of aldehydes (Al‐Bayati and Mohammed 2009). Compared to the other G‐EC spectra, the spectrum of G‐EC‐4 exalted the signal (several bands) in the region 2160–2000 cm^−1^, corresponding to overtone and combination bands of the substituted benzene ring. Moreover, new sharp peaks appeared at 1797, 1736, and 1660 cm^−1^. The band at 1797 cm^−1^ is assigned to C═O stretch of carbonyl compounds, possibly esters, aldehydes, ketones, found as minor co‐products of the TCA product. The band at 1736 cm^−1^ is ascribed to the C═O stretch of aldehydes (Al‐Bayati and Mohammed 2009; Liu et al. 2017; Lixourgioti et al. 2022). The band at 1660 cm^−1^ is related to the C═C stretch of alkenes overlapped with the Amide I band of G proteins and potentially the C═N stretch of the imine group (assuming an attachment reaction between proteins and TCA occurred). This band is critical because the aldehyde group of TCA is expected to react with the amino groups of G to form covalent amide and imine bonds through Schiff base reaction. Additionally, the Michael reaction takes place, which involves the bonding of the amino groups of G with the α,β‐unsaturated molecules of TCA (Thongsrikhem et al. 2022).

The notably sharp band at 1371 cm^−1^ is related to H─C═O bending of the aliphatic aldehyde chain in TCA. Two (2) other bands at 748, and 693 cm^−1^ (far infrared region) are more finely related to the structure of TCA and are respectively attributed to C─H out‐of‐plane bending of the monosubstituted and *m*‐disubstituted aromatic rings of TCA. Kenawy et al. (2019) also reported similar peaks for G‐chitosan membranes containing TCA. The *m*‐disubstitution of the benzene ring of TCA is in accordance with an assumed Michael reaction. Consequently, this analysis suggests that TCA can establish inter‐ and intramolecular covalent bonds and create an interpenetrating crosslinked network between G chains. Tengroth et al. (2005) reported a similar frequency range (1800–800 cm^−1^) corresponding to crosslinked G capsules with some aldehydes such as formaldehyde, acetaldehyde, and propionaldehyde compared to pure G capsules.

In correlation with these FTIR results, and regarding film microstructure, some studies also revealed a typical surface topography and cross‐section of crosslinked G films by SEM analysis. Previously in our laboratories, we have observed similar microstructures on crosslinked G‐TCA films, with a rough, compact surface and a wavy cross‐section, due to the dispersion (and possible aggregation) of hydrophobic TCA into the hydrophilic G matrix (Jaiswal et al. 2024). Besides, Choi et al. (2018) observed a more compact structure of the films (reduction of pores and increase of aggregation) via intermolecular interactions after crosslinking by oxidized phenolic compounds (tannic acid, caffeic acid, and green tea extract). They also reported a rougher surface, possibly due to the hydrophobicity and lower solubility of phenolic compounds, but also to covalent/non‐covalent bonding between proteins and phenolics.

The possible mechanisms of attachment/crosslinking are originally based on that described in the presence of formaldehyde and G by Ofner et al. (2001), Tengroth et al. (2005), or Azeredo and Waldron (2016). The interaction between aldehyde and G typically begins with the formation of an amine methylol group (i.e., amine hydroxymethyl) intermediate, primarily from lysine and arginine groups within G. Subsequently, crosslinking may occur between lysine and arginine or between arginine and arginine residues via a methylene bridge. Otherwise, the reaction mechanism of the crosslinking of G by TCA has been reported by several authors (Balaguer et al. 2011; Thongsrikhem et al. 2022; Jaiswal et al. 2024). Different reaction pathways are governed by (i) the formation of a Schiff base (imine bond) between the aldehyde groups of TCA and the primary amino groups of G (e.g., from the lysine moiety) via condensation reaction, and (ii) the formation of a methanetriyl group (─CH<) via Michael 1,4 addition of the amino groups of G on the α,β‐unsaturated bond of TCA.

Regarding G‐EC interactions, it was reported that G integrates through hydrogen bonding with bacterial cellulose in the composite film of bacterial cellulose and G (Chang et al. 2012). Similarly, Liu et al. 2018c, 2018d) reported that hydrogen bonding interactions occurred in G‐EC composite nanofiber. The molecular structure of the composite film is physically intertwined and held together by hydrogen bonds. These bonds create a network‐like structure, improving the material's strength, stability, and hydrophobicity. Composites containing a higher content of EC have a hydrophobic surface, low water vapor permeability, and strong water resistance, helping to prevent food surface dehydration.

### 3.7 in vitro Antimicrobial Test

**FIGURE 4:**
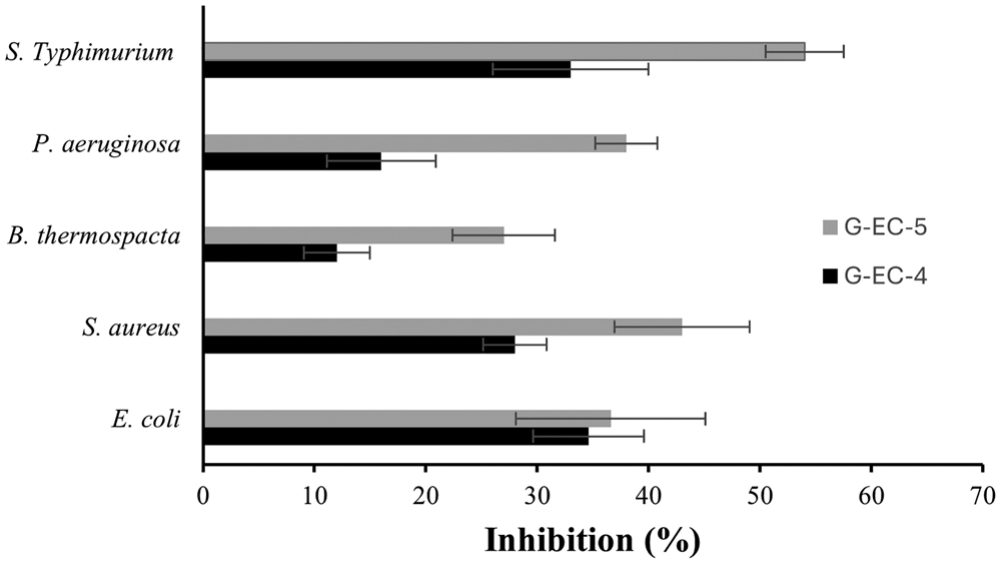
Inhibition activity of nanocomposite film against selected bacteria.

To assess the antimicrobial properties of the active films, various contaminants, and pathogens commonly found in the food industry, including *S*. Typhimurium, *P. aeruginosa*, *B. thermosphacta*, *S. aureus*, and *E. coli* were tested. Films without TCA and AgNPs had no antimicrobial activity (0% inhibition). The results, depicted in Figure 4, revealed that the G‐EC‐4 film had a significant antimicrobial activity against all bacteria compared to the control film (*p* ≤ 0.05). Notably, the film containing TCA and AgNPs (G‐EC‐5) was significantly more effective (*p* ≤ 0.05) than the film only incorporated with TCA (G‐EC‐4), except for *E. coli* (*p* > 0.05). Indeed, the G‐EC‐5 film had the highest antimicrobial activity against *S*. Typhimurium with an inhibition of 54%, followed by *S. aureus*, *P. aeruginosa*, *E. coli*, and *B. thermosphacta* with inhibition capacities of 43%, 38%, 36%, and 27%, respectively. The same trend was obtained for the G‐EC‐4 film. Previous studies have reported *Listeria* growth inhibition at a concentration of 125–250 µg/mL of TCA (Mith et al. 2014; Kerekes et al. 2019). Besides, AgNPs penetrate the bacterial cell wall and interact with cellular ingredients, leading to cell death. Overall, the incorporation of TCA (in G‐EC‐4 and G‐EC‐5 films) and AgNPs (in G‐EC‐5 film) exhibited strong antimicrobial activity against the tested bacteria. This is mainly attributed to the lipophilic nature of TCA, allowing it to interact with the bacterial cell membrane, potentially altering its permeability, which may lead to the release of intracellular compounds from the bacterial cytoplasm (Mahmud et al. 2022). Ahmed et al. (2018) evaluated the in vitro antimicrobial activity of LLDPE film containing cinnamon EO and Ag‐Cu NPs. They have found that the bioactive film could reduce the growth of *S*. Typhimurium, *Campylobacter jejuni*, and *Listeria monocytogenes*. Phaiju et al. (2020) showed that electrospun fibers containing cinnamic acid (CA) were successful in inhibiting the growth of both gram‐positive and gram‐negative bacteria. This inhibition may result from CA causing enzyme inactivation or protein denaturation. Furthermore, Liu et al. (2021) reported that the antimicrobial activity of thymol and carvacrol was related to the leakage of phosphate ions through *P. aeruginosa*. In food applications on meat products, Shahbazi and Shavisi (2018) obtained results like our study with bioactive nanocomposite chitosan‐CNPs‐EPE films against pathogens. They measured significant inhibition zones increasing with CNP and EPE concentration (up to 2%). Moreover, they found that the lowest bacterial population in an in situ test of 14 days was for samples treated with CH + CNPs 2% + EPE 2% (highest content of nanoparticle and bioactive agent).

### 3.8 in situ Antimicrobial Test

**FIGURE 5:**
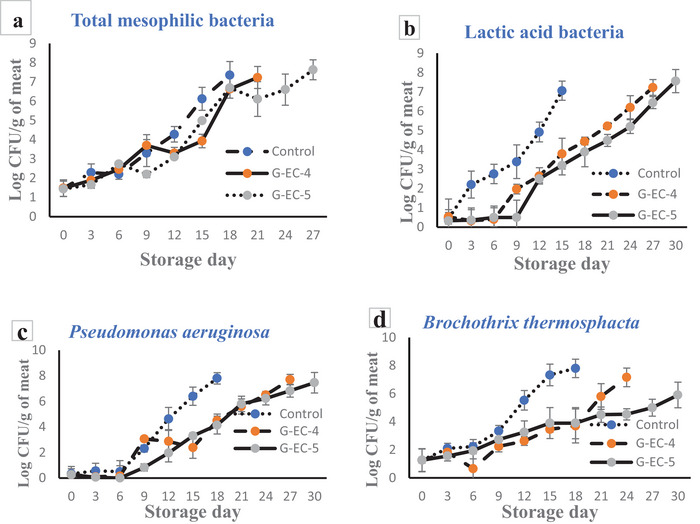
Evolution of the microbial counts of meat (a) TMF, (b) LAB, (c) *P. aeruginosa*, and (d) *B. thermosphacta*) treated with the bioactive films G‐EC‐4 and G‐EC‐5, during storage at 4°C.

TMF counts are an important indicator of shelf‐life and contamination of fresh beef meat (Shahbazi and Shavisi 2018). The TMF counts on refrigerated sliced meat packaged with antimicrobial active films (G‐EC‐4 and G‐EC‐5) are presented in Figure 5. At the beginning of the experiment, the count of TMF was 1.35–1.44 log CFU/g in all groups (*p* > 0.05). During storage, the TMF value in all the groups increased significantly (*p* ≤ 0.05). The control group (meat without film) exhibited the fastest growth in TMF during storage and had significantly higher values compared to the groups wrapped with active films from day 6 to the end of storage time (*p* ≤ 0.05). The acceptable limit of TMF for fresh meat was considered as 7 log CFU/g. The TMF values of the control group exceeded this threshold after 18 days of storage, while the G‐EC‐4 and G‐EC‐5 groups reached the threshold after 21 and 27 days, respectively. The TMF value of the G‐EC‐5 group was significantly lower than the control and G‐EC‐4 groups from day 6 to the end of storage time (*p* ≤ 0.05). This indicates that the combination of TCA and AgNPs was more powerful in inhibiting the growth of bacteria, as indicated by the results of the in vitro test.

LAB can grow under high concentrations of CO_2_ and are responsible for the reduction of meat pH, contributing to meat spoilage and subsequent organoleptic downgrading of meat products. *Pseudomonas* spp. and *B. thermosphacta* are also considered spoilage microorganisms of aerobically stored meat products in refrigerated conditions (Shahbazi and Shavisi 2018; Pothakos et al. 2015). At day 0, the counts of LAB, *P. aeruginosa*, and *B. thermosphacta* were in the ranges of 1.30–1.91, 0.20–0.53, and 1.11–1.56 log CFU/g, respectively. Hence, these counts were below the detection level for meat (*p* > 0.05), indicating the appropriate quality of meat. The counts for all bacteria increased significantly (*p* ≤ 0.05) over time during the storage period in all groups, although for the control at a higher rate than the treatment groups. A significant antimicrobial effect of TCA and TCA‐AgNPs was noticed (*p* ≤ 0.05) in the counts of all bacteria during the storage conditions. Indeed, the control passed 7 log CFU/g for LAB on day 15, while the G‐EC‐4 and G‐EC‐5 groups reached 7.23 and 7.56 log CFU/g on days 27 and 30, respectively (extension by 13 and 15 days). As a similar trend, the control reached 7 log CFU/g for *P. aeruginosa* and *B. thermosphacta* on day 18 of the storage period. On the other hand, the G‐EC‐4 and G‐EC‐5 groups passed 7 log CFU/g on days 27 and 30 for the count of *P. aeruginosa* (extension by 9 and 12 days) and days 24 and 30 for the count of *B. thermosphacta* (extension by 6 and 12 days), respectively. A significantly greater antimicrobial activity in the G‐EC‐5 group (*p* ≤ 0.05) was ascribed to the synergistic effect generated by the simultaneous release of TCA and silver ions from the composite film. This synergistic action disrupted metabolic processes and caused damage to the bacterial cell wall, ultimately leading to cell death.

The migration of AgNPs occurs through 2 mechanisms: detachment of the AgNPs from the nanocomposites and the dissolution of silver ions due to oxidation. In addition, a combination of EC and G reduced the moisture permeability of the film due to the formation of a compact structure through hydrogen bonding. This, in turn, enhances the antibacterial activity of the coating, making it more effective in preventing moisture infiltration and inhibiting bacterial growth (Mahmud et al. 2022). Therefore, based on these results, TMF growth was a limiting factor, and the shelf‐life of the sliced meat was extended by 3 days and 9 days in groups G‐EC‐4 and G‐EC‐5, respectively, more than the control group (*p* ≤ 0.05). In agreement with our findings, Shahbazi and Shavisi (2018) reported an important reduction of TMF, LAB, and *Pseudomonas* spp. in minced beef meat stored at 4°C when applying their chitosan‐CNPs‐EPE packaging film. Indeed, samples treated with CH + CNPs 2% + EPE 2% induced a significant shelf‐life extension by 14 days, reducing TMF, LAB, and *Pseudomonas* counts. This extension of shelf‐life with a biopolymer‐based film containing bioactive extracts did not negatively affect the sensory properties (odor, color, overall acceptability) of meat. In a previous study conducted in our laboratories, Mahmud et al. (2024a) characterized G‐based crosslinked films containing a bioactive formulation of plant extracts. Regarding meat evaluation, they studied the effect of the films on the microbiological quality and color of the meat, as well as their ability to prevent lipid oxidation via the quantification of thiobarbituric acid reactive substances (TBARS). In combination with modified atmosphere packaging (MAP), they preserved meat redness (a* parameter > 14.5 at day 20, as a limit of acceptability), prevented lipid oxidation (< 2 mg malondialdehyde/kg), and extended the shelf‐life up to 26 days. In another similar application on refrigerated meat, Goudarzi et al. (2023) showed that their KC‐PVA electrospun fiber mats (containing plum extract and EGCG) had good antimicrobial properties by extending the shelf‐life by 10 days (from 5 to 15 days, referring to total viable counts) and a high degree of appreciation in sensory properties (including meat color). In correlation with these results, they reported that the pH, peroxide value (PV; related to lipid primary oxidation), and total volatile basic nitrogen (TVB‐N; related to spoilage microorganisms) were also reduced, demonstrating a better quality of meat throughout storage.

As discussed earlier, this interaction of TCA and G can be seen by the formation of Schiff bases and/or Michael adducts. In this process, the amine groups of G react with the aldehyde group of TCA, leading to the creation of hydrophobic regions within the membrane matrix. Consequently, this enhances the interaction between the compound and the peptidoglycan of the cell wall and the lipoprotein in the outer membrane, facilitating its attachment to the cell wall of microorganisms (Kenawy et al. 2019). Moreover, the inhibitory effect of TCA is attributed to the interaction between positively charged amine groups and the negatively charged cell surface of bacteria, which induces significant alterations in cell wall permeability. This interaction ultimately results in the blocking of channels responsible for the exchange of electrolytes and nutrients. Consequently, intracellular substances such as electrolytes, proteins, amino acids, glucose, and lactic dehydrogenase leak from the bacterial cells. This disruption leads to the inhibition of the normal metabolic processes in microorganisms, ultimately culminating in the death of bacterial cells (Kenawy et al. 2019; Masoomian et al. 2023).

As reported above, a mechanism of action describing the antimicrobial effects of AgNPs and other metal NPs was reported by Mahmud et al. (2022): (i) either via their dissolution under the form of free metal ions; (ii) or via oxidative stress generated by reactive oxygen species (ROS) and associated free radicals, and facilitated by electrostatic interactions between AgNPs (positively charged) and the bacterial membrane (negatively charged).

## 4 Conclusion

In this study, a nanocomposite film composed of G and EC was successfully formulated with an optimized biopolymer content to overcome the limitations of the pure G film. Specifically, the optimized G‐EC‐3 film (1:2 w/w) significantly reduced WS_C_ from 57.9% (pure G film) to 7.8% and reduced WVP and mechanical properties. The addition of EC to the polymer blend significantly improved the stability of the polymer network via hydrogen bonding with the protein chains. Subsequently, in the formation of G‐EC‐4 films, the crosslinking agent TCA enhanced the film's structural integrity, creating an amide/imine bond‐ stabilized matrix with a 33% degree of crosslinking, enhanced physicochemical properties, and strong antibacterial properties. These results highlighted the double role of TCA as both a crosslinker and a bioactive agent. Crosslinking improved mechanical properties and WVP, while further reducing the film's WS, parameters essential for food packaging applications. Afterwards, in the formation of G‐EC‐5 films, the incorporation of AgNPs with TCA enhanced the antimicrobial properties of the film, inhibiting pathogenic bacteria such as *S*. Typhimurium, *E. coli*, *S. aureus, P. aeruginosa*, and the spoilage bacterium B. *thermosphacta*. In addition to these biological properties, the incorporation of AgNPs induced a higher degree of crosslinking (57%) and improved the physicochemical properties of the film, with a very low WSc of 0.33%, a low WVP of 2.39 g·mm·m^−^^2^·day^−^^1^·kPa^−^^1^, a higher TS of 9.6 MPa, a higher *T*_max_ of 370°C–380°C, and a *T*_g_ of 29.5°C.

In conclusion, the resulting G‐EC‐5 nanocomposite film exhibited improved structural stability, mechanical resilience, and antibacterial efficacy. Testing conducted on refrigerated sliced meat using these bioactive films showed a notable extension in shelf‐life: 3 days for the G‐EC‐4 film and 9 days for the G‐EC‐5 film, thus demonstrating their value as biodegradable and antimicrobial packaging. This study therefore highlights the potential of G‐EC‐based films as a biobased active food packaging solution for preserving the quality and extending the freshness of perishable foods.

## Author Contributions

**Elham Sarmast**: conceptualization, data curation, formal analysis, investigation, methodology, software, writing – original draft. **Shiv Shankar**: methodology, resources, validation. **Stephane Salmieri**: methodology, resources, validation, visualization, writing – review and editing. **Sahra Amel Rahmouni**: formal analysis, software, investigation. **Monique Lacroix**: conceptualization, funding acquisition, project administration, resources, supervision, validation, writing – review and editing.

## Conflicts of Interest

The authors declare no conflicts of interest.

## Supporting information




**Supplementary Figure S1**: jfds70851‐sup‐0001‐FigureS1.docx

## Data Availability

Data are available upon request.
