# Supplementary material for: Development of an Active Crosslinked Nanocomposite Film Based on Gelatin‐Ethyl Cellulose, Trans‐Cinnamaldehyde, and Silver Nanoparticles for the Preservation of Sliced Meat
Source: J Food Sci. 2026 Jan 16;91(1):e70851. doi: 10.1111/1750-3841.70851 (PMC12811702; doi:10.1111/1750-3841.70851)
Supplement: Supplementary file 1 — Supplementary Figure S1: jfds70851‐sup‐0001‐FigureS1.docx [file JFDS-91-0-s001.docx]

**Supplementary data**


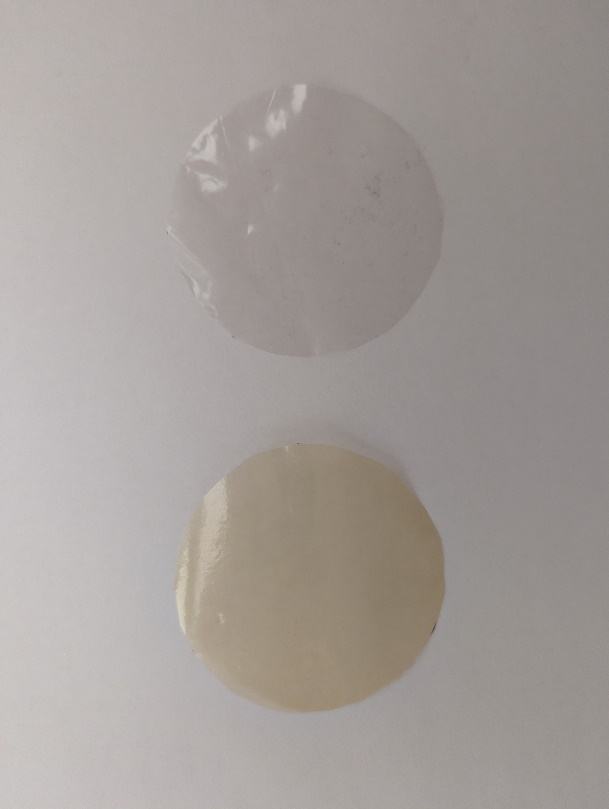


**a**

**b**

**Figure 1.** Digital images of polymer blend G-EC-3 film (**a**) and bioactive nanocomposite G-EC-5 film containing TCA and AgNPs (**b**).
